# Human neutrophilic cells express anti-inflammatory EBI3 in response to *Neisseria meningitidis*

**DOI:** 10.1016/j.jneuroim.2026.578995

**Published:** 2026-06-01

**Authors:** Andrew M. Dunphy, Quinton A. Krueger, M. Brittany Johnson, Ian Marriott

**Affiliations:** a Department of Biological Sciences, University of North Carolina at Charlotte, Charlotte, NC 28262, USA; b Computational Intelligence for Predicting Health and Environmental Risks (CIPHER) University of North Carolina at Charlotte, Charlotte, NC 28262, USA

**Keywords:** Human, Neutrophils, Astrocytes, *Neisseria meningitidis*, EBI3, Anti-inflammatory

## Abstract

During bacterial infections of the central nervous system (CNS), the glia-neutrophil axis can drive a vicious cycle that results in lethal neuroinflammation. However, it is becoming apparent that glia can also be a significant source of immunosuppressive factors following bacterial challenge, presumably to mitigate inflammatory CNS damage. Similarly, neutrophils are known to express anti-inflammatory mediators following activation but their ability to produce them in response to clinically important neurotropic bacteria is poorly understood. In the present study, we have characterized the responses of a differentiated human neutrophil cell line to *Neisseria meningitidis* challenge by RNA Tag-Seq analysis and show marked enrichment in the expression of genes associated with Toll-like receptor-mediated NF-κB and AP-1 signaling, and inflammatory gene products. Interestingly, we also describe their upregulated expression of EBI3, a molecule that can exert immunosuppressive effects outside of its role as a component of IL-27 and IL-35. We have confirmed that these neutrophils produce and secrete EBI3 following *N. meningitidis* challenge, and we have begun to assess the functional ramifications of such production with the demonstration that EBI3 can significantly reduce the production of CXCL8 by infected primary human astrocytes. These results raise the possibility that neutrophils recruited to the CNS during *N. meningitidis* infection produce EBI3, thereby limiting inflammatory glial responses to this neurotropic bacterium.

## 1. Introduction

The inflammatory damage associated with bacterial infection of the central nervous system (CNS) is driven by the responses of both resident glia and recruited leukocytes, most notably neutrophils. Glia react to neurotropic bacteria, such as *Neisseria meningitidis*, by producing immune mediators that impact blood-brain barrier integrity and recruit neutrophils (Ampie and McGavern, 2022). While these leukocytes function to eliminate bacteria, they also exacerbate neuroinflammation and recruit more neutrophils. As such, this glia-neutrophil axis can foster a vicious cycle that results in CNS damage (Dunphy and Marriott, 2026). However, bacterially challenged glia can also express immunosuppressive mediators including IL-10 family members, TGF-β, and ISG15 (Rasley et al., 2006; Welser-Alves and Milner, 2013; Burmeister and Marriott, 2018; Dunphy et al., 2026), suggesting a negative feedback loop that mitigates inflammation. Similarly, neutrophils can produce anti-inflammatory factors but their ability to respond to neurotropic bacteria in this manner is poorly studied.

Here, we report the upregulated expression of inflammatory and leukocyte migration-associated genes in human neutrophils following *N. meningitidis* challenge, with marked enrichment in those associated with TLR-mediated inflammation. Interestingly, we also describe the upregulated expression of Epstein-Barr virus induced gene 3 (EBI3), which can exert immunosuppressive effects (Takeda et al., 2014; Hildenbrand et al., 2023). We have confirmed that *N. meningitidis* challenged neutrophils produce EBI3 and begun to assess its significance with the demonstration that it can reduce bacteria-induced neutrophil-recruiting chemokine production by primary human astrocytes. These results suggest that EBI3 produced by neutrophils could serve to limit the neuroinflammatory damage associated with this neurotropic bacterium.

## 2. Materials and methods

### 2.1. Primary human astrocytes and neutrophil cell line

Human leukemia-60 cells (HL-60; ATCC) were differentiated to mature neutrophil-like cells (CD11b/CD35^hi^ CD71^lo^) and maintained as described (Sipprell et al., 2023). Primary human cortical astrocytes were purchased and cultured in the media supplied by the vendor (ScienCell Research Laboratories). In some studies, cell viability was assessed by a colorimetric assay (CellTiter 96^®^ AQueous, Promega).

### 2.2. N. meningitidis propagation and human cell infection

*N. meningitidis* strain MC58 (ATCC) was cultured as described (Rasley et al., 2006). The number of colony forming units were determined by spectrophotometry and human cells were infected at multiplicities of infection (MOI) ranging from 10 to 50 bacteria to each human cell in antibiotic-free medium for 2 h at 37 °C with 5% CO_2_. While a single bacterial strain was used in these studies, this strain was selected as it is a well-characterized, hypervirulent strain of serogroup B that is a major cause of bacterial meningitis. The doses employed are based on bacterial numbers in the cerebral spinal fluid during bacterial meningitis (Bingen et al., 1990). After 2 h post-infection (hpi), 1% penicillin-streptomycin was added to kill extracellular bacteria.

### 2.3. RNA Tag-Seq analysis

RNA was isolated using a GeneJET RNA purification kit. The University of Texas at Austin (GSAF) prepared Tag-Seq libraries as described (Meyer et al., 2011) and sequenced libraries using an Illumina HiSeq 3000. The 100-bp, single-end reads were trimmed and quality-filtered using cutadapt v2.6 (Martin, 2011), which were mapped to the reference genome (GRCh38.p14) using Bowtie2 v2.5.3 with options –no-hd –no-sq –no-unal -k 5 (Langmead and Salzberg, 2012). Differential expression and outlier analyses were performed using DESeq2 (Love et al., 2014) and ArrayQualityMetrics (Kauffmann et al., 2009), respectively. Normalized and rlog-transformed counts were used in all downstream analyses (R3.5.0; R Core Team, 2015). Pairwise comparisons between treatments using Wald tests in DESeq identified significantly differentially expressed genes (DEGs) with a false-discovery rate (FDR; Benjamini-Hochberg). A log2fold change > |2| and adjusted *P*-value [padj] < 0.05 was considered significant. Significantly up-regulated genes were imported into ShinyGO 0.85 (Ge et al., 2020). Significantly enriched gene ontology (GO) terms and the KEGG pathway for TLRs (map04620) were stylized using the Adobe suite (Kanehisa and Goto, 2000).

### 2.4. Immunoblot analyses

Cell lysates were evaluated using a rabbit polyclonal antibody against human EBI3 (Abcam; ab307195) and a horseradish peroxidase-conjugated secondary anti-rabbit antibody (Cell Signaling), and detected with a West Pico PLUS ECL kit (Thermo Scientific) as described (Furr et al., 2008). Blots were re-probed with a rabbit monoclonal antibody against β-actin (Cell Signaling; 4967S) to assess protein loading. Human U937 cells differentiated to a monocytic phenotype with DMSO (Myers and Siegel, 1984) were used as a positive control. Immunoblots shown are representative of at least three separate experiments and imaged using an Azure 300 with AzureSpotPro software for densitometric analysis.

### 2.5. ELISAs

Commercially available ELISA kits (R&D Systems) were employed to measure EBI3 and CXCL8 production according to the manufacturer's directions.

### 2.6. Statistical analysis

Data is expressed as the mean ± standard error of the mean (SEM). GraphPad Prism was used to conduct statistical analyses including one-way and two-way analysis of variance (ANOVA) with Šidák’s multiple comparisons test, where *p* < 0.05 was considered statistically significant.

## 3. Results

### 3.1. Upregulated EBI3 expression in N. meningitidis challenged human neutrophilic cells

**Fig. 1.**
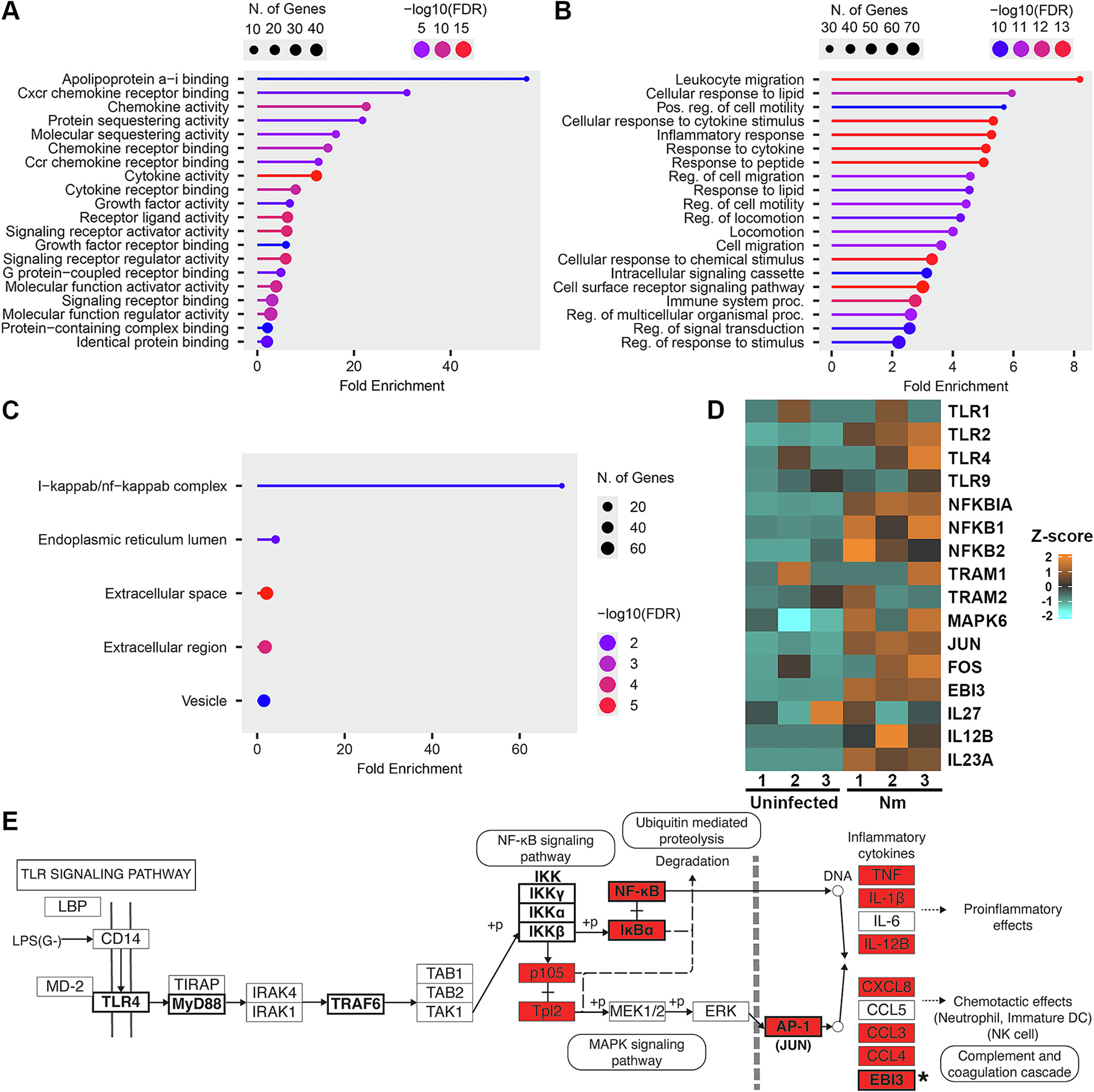
*N. meningitidis*-induced mRNA expression in human neutrophilic cells. Cells were uninfected or infected with *N. meningitidis* (MOI of 10) and RNA isolated at 2h for Tag-Seq analysis. Panels A-C: Gene ontology lollipop charts displaying fold enrichment for molecular functions (A), biological processes (B), and cellular components (C). Point size represents the number of enriched genes and color indicates −log_10_(FDR). Panel D: heatmap displaying DEGs encoding immune mediators in uninfected and infected (Nm) neutrophils (*n* = 3). Color key describes the *Z*-score. Panel E: log_2_fold > 2, p adj<0.05 upregulation of genes associated with TLR4-mediated signaling adapted from KEGG pathway (map04620). Enriched genes are indicated in red.

RNA Tag-Seq analysis identified 171 DEGs in *N. meningitidis* challenged human neutrophilic cells. Gene ontology analysis of infected neutrophils showed 15+ fold enrichment of gene products associated with chemokine signaling (Fig. 1 A), and significant enrichment in those associated with leukocyte migration, inflammation, and TLR-mediated NF-κB signaling (Fig. 1B–C). Interestingly, these cells showed a marked increase in levels of mRNA encoding EBI3 (Fig. 1D), a component of IL-27 and IL-35 that are members of the IL-12 family of heterodimeric cytokines. Furthermore, EBI3 upregulation is consistent with KEGG pathway analysis, which supports induction of mRNA encoding immune mediators linked to TLR signaling (Fig. 1E) (Kanehisa and Goto, 2000). However, while the p40 subunit of IL-12 also showed upregulation following *N. meningitidis* infection, the p28 subunit of IL-27 did not (Fig. 1D) and mRNA encoding the p35 subunit of IL-35 (*IL-12a*) was undetectable (data not shown) suggesting that EBI3 expression in these cells is independent of these heterodimers.

### 3.2. Neisseria meningitidis-challenged human neutrophils produce EBI3, thereby limiting inflammatory responses of human astrocytes

**Fig. 2.**
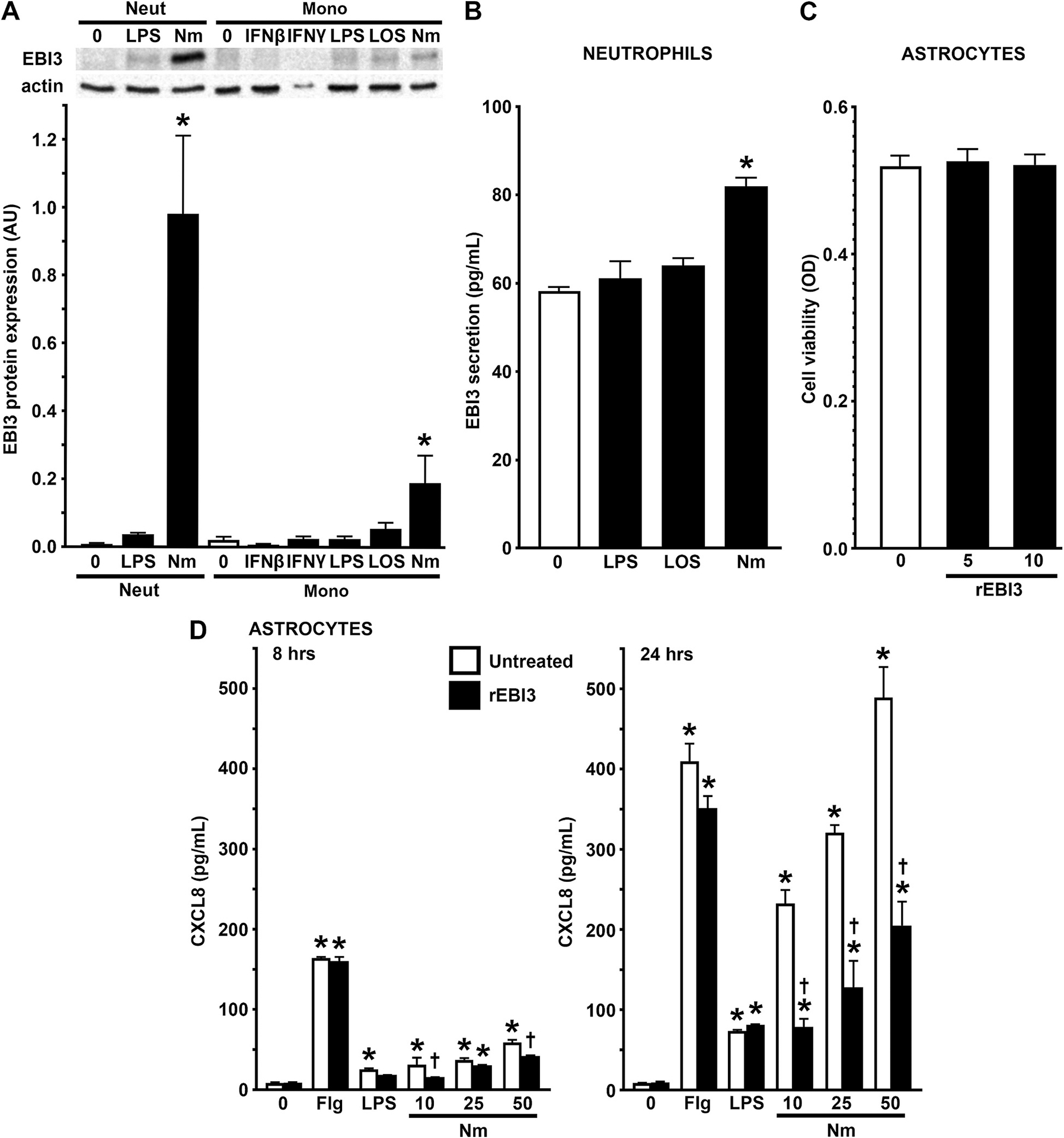
*N. meningitidis* challenged human neutrophils produce EBI3, which can attenuate the neutrophil-chemoattractant responses of primary human astrocytes. Panels A-B: neutrophilic cells (Neut) and differentiated U937 monocyte-like cells (Mono) were untreated (0), or exposed to IFN-β (1 ng/mL), IFN-γ (10 ng/mL), LPS (10 and 100 ng/mL for Neut and Mono, respectively), *N. meningitidis*-derived LOS (100 ng/mL) or *N. meningitidis* (MOI of 50) for 24 h prior to immunoblot analysis for EBI3 and β-actin (A) and EBI3 release by ELISA (B). Relative EBI3 protein expression was determined by densitometric analysis normalized to untreated cells. Data shown is the mean +/− SEM (n = 3). Asterisks indicate a significant difference from untreated cells (one-way ANOVA; *p* < 0.05). Panel C: Astrocytes were untreated (0) or treated with rEBI3 (5 and 10 ng/mL) for 24 h prior to cell viability assessment (OD 490 nm). Panel D: Astrocytes were untreated (0) or challenged with flagellin (Flg, 25 ng/mL), LPS (10 ng/mL), or *N. meningitidis* (MOI of 10, 25, and 50) plus or minus rEBI3 (10 ng/mL) for 8 or 24 h. Data shown is mean +/− SEM of at least 2 independent experiments and asterisks and daggers indicate significant differences from untreated cells and similarly challenged cells, respectively (two-way ANOVA; p < 0.05).

We have confirmed that *N. meningitidis* challenge of human neutrophilic cells results in marked EBI3 protein production at 24 hpi at levels substantially greater than those seen in similarly challenged human monocytic cells (Fig. 2 A). Interestingly, LPS and LOS treatment both elicited modest EBI3 protein expression by neutrophils and monocytes (Fig. 2 A). Furthermore, we have confirmed that *N. meningitidis* challenge can evoke demonstrable EBI3 secretion by human neutrophils (Fig. 2B).

To begin to determine the functional significance of EBI3 production by *N. meningitidis* challenged neutrophils, we have assessed the effect of recombinant EBI3 (rEBI3; Abcam ab83026) on *N. meningitidis*-induced human glial responses at concentrations (5–10 ng/mL) previously reported to exert *in vitro* effects (Hildenbrand et al., 2023). As shown in Fig. 2C, treatment of astrocytes with rEBI3 for up to 24 h had no effect on cell viability. However, rEBI3 treatment at 10 ng/mL markedly reduced *N. meningitidis*-induced CXCL8 production by these cells as rapidly as 8 hpi (Fig. 2D). In addition, we have found that 5 ng/mL rEBI3 tended to decrease CXCL8 release by *N. meningitidis* infected cells (MOI of 25), although this effect was not statistically significant (data not shown). Interestingly, these effects were restricted to responses to this bacterium as rEBI3 did not significantly affect CXCL8 release induced by TLR4 or TLR5 agonists (Fig. 2D). These findings support the notion that neutrophil-derived EBI3 could limit detrimental inflammation attributable to this neurotropic bacterium.

## 4. Discussion

Glia have an important role in the recognition of bacterial pathogens, such as *N. meningitidis*, and the production of chemotactic factors that preferentially recruit neutrophils (Dunphy and Marriott, 2026). Once recruited, these cells respond to bacteria in a manner that augments glial inflammatory responses and promotes further neutrophil recruitment creating a vicious cycle (Dunphy and Marriott, 2026). Here, we have characterized the responses of human neutrophilic cells to *N. meningitidis* challenge. Our RNA Tag-Seq analysis shows the upregulation of genes involved in inflammation and leukocyte migration and enrichment of genes associated with TLR-mediated NF-κB and AP-1 signaling, and inflammatory gene products. It should be noted that previous studies have reported broader transcriptional responses of primary human neutrophils to LPS challenge (Ismailova et al., 2023). This is likely attributable to the cell line employed here, which has been shown to express lower levels of TLR4 and NF-kB/TRIF signaling, incomplete differentiation and physiological priming, and differences in gene promoter and enhancer accessibility than primary neutrophils (Yaseen et al., 2017; Mark Welch et al., 2017; Rincón et al., 2018).

However, *N. meningitidis* can also induce the production of anti-inflammatory mediators by glia and neutrophils (Pollard et al., 1999; Cooley et al., 2014; Burmeister and Marriott, 2018; Dunphy et al., 2026) suggesting that glia-neutrophil crosstalk can mitigate runaway CNS inflammation (Dunphy and Marriott, 2026). EBI3 is a subunit of IL-27 and IL-35, both of which have immunosuppressive effects (Ye et al., 2021). In addition, evidence is accumulating that EBI3 may exert IL-27 and IL-35 independent anti-inflammatory effects (Takeda et al., 2014; Hildenbrand et al., 2023). Our transcriptional analysis revealed *N. meningitidis*-induced EBI3 mRNA expression in human neutrophils but not the p28 and p35 subunits of IL-27 and IL-35, respectively. Importantly, we have confirmed that these cells show robust EBI3 protein expression and demonstrable secretion following *N. meningitidis* challenge. This finding is consistent with a study demonstrating EBI3 production by human neutrophils in the absence of IL-27 or IL-35 in response to TLR8 agonists or TNF (Cassatella et al., 2020). Furthermore, we have found that LPS elicited only modest EBI3 protein production by neutrophils and both LPS and LOS failed to elicit its secretion, which agrees with the prior demonstration that TLR4 ligands are relatively weak stimuli for human neutrophil EBI3 expression (Cassatella et al., 2020).

To begin to determine the significance of *N. meningitidis*-induced production of EBI3 by neutrophils, we evaluated the effect of EBI3 on the responses of primary human glia to this neurotropic bacterium. We show that rEBI3 treatment significantly reduces production of CXCL8, a potent neutrophil-attracting chemokine, by *N. meningitidis* challenged astrocytes. Importantly, these effects appear to be specific and direct as rEBI3 failed to affect either TLR4 or TLR5-induced responses and occurred without changes in cell viability. Together, these results raise the intriguing possibility that neutrophils recruited to the CNS during *N. meningitidis* infection produce EBI3 to limit glial immune functions, presumably to limit the inflammatory damage associated with such infections.

## Data Availability

Tag-Seq generated reads are available at the NCBI SRA repository under the BioProject PRJNA1176688. The data used and/or analyzed during the current study available from the corresponding author on reasonable request.
